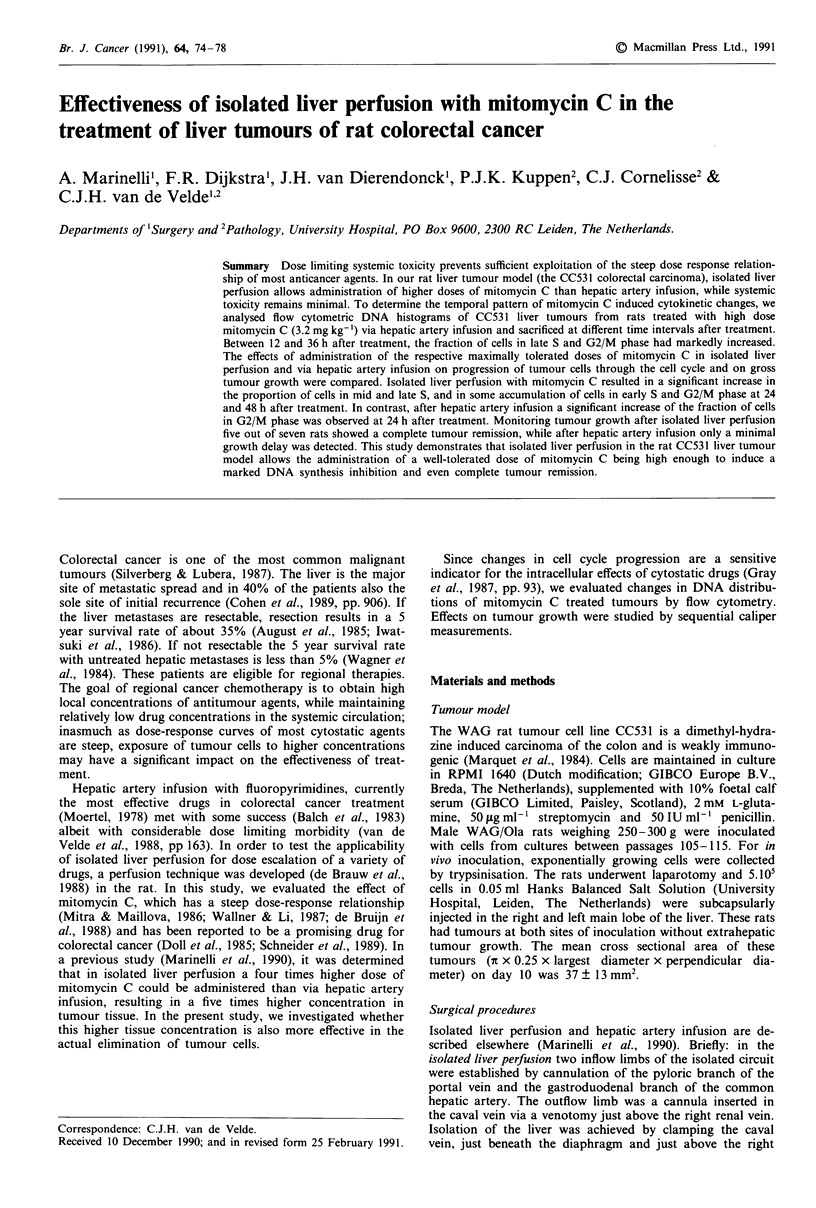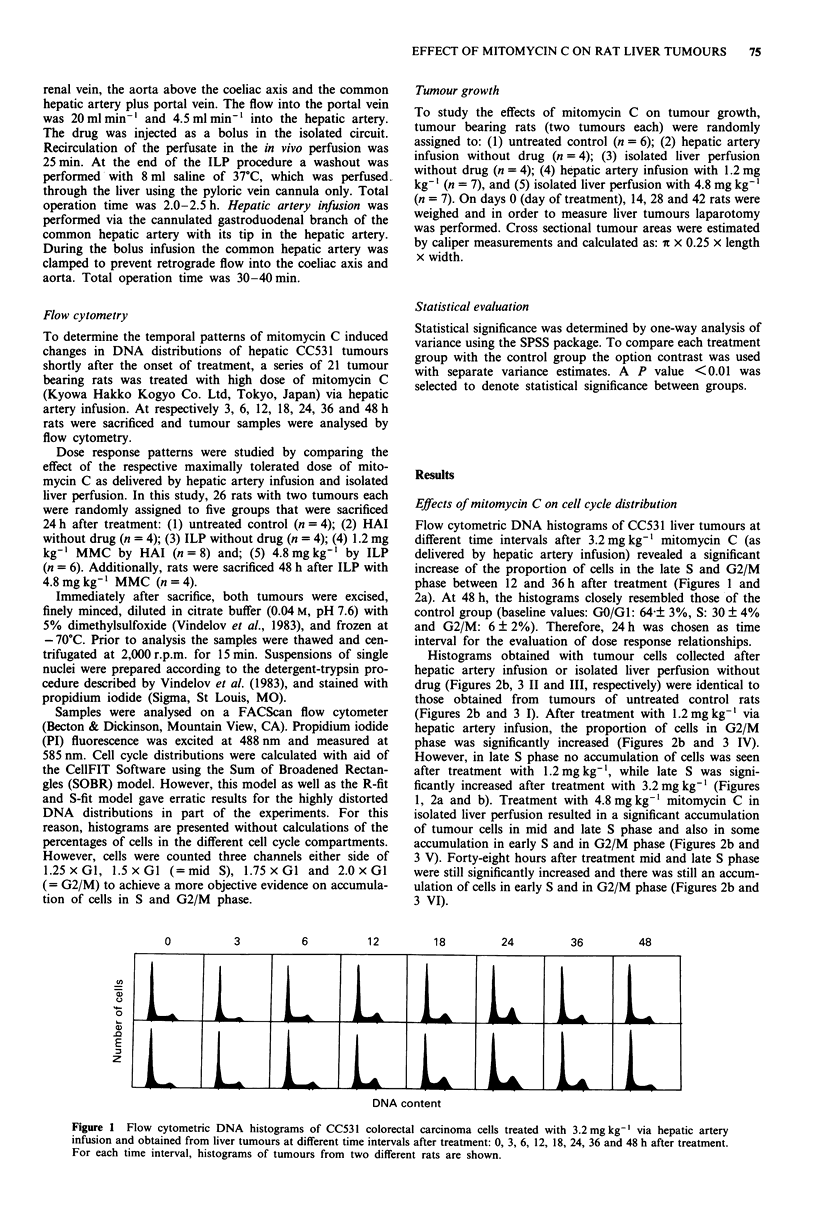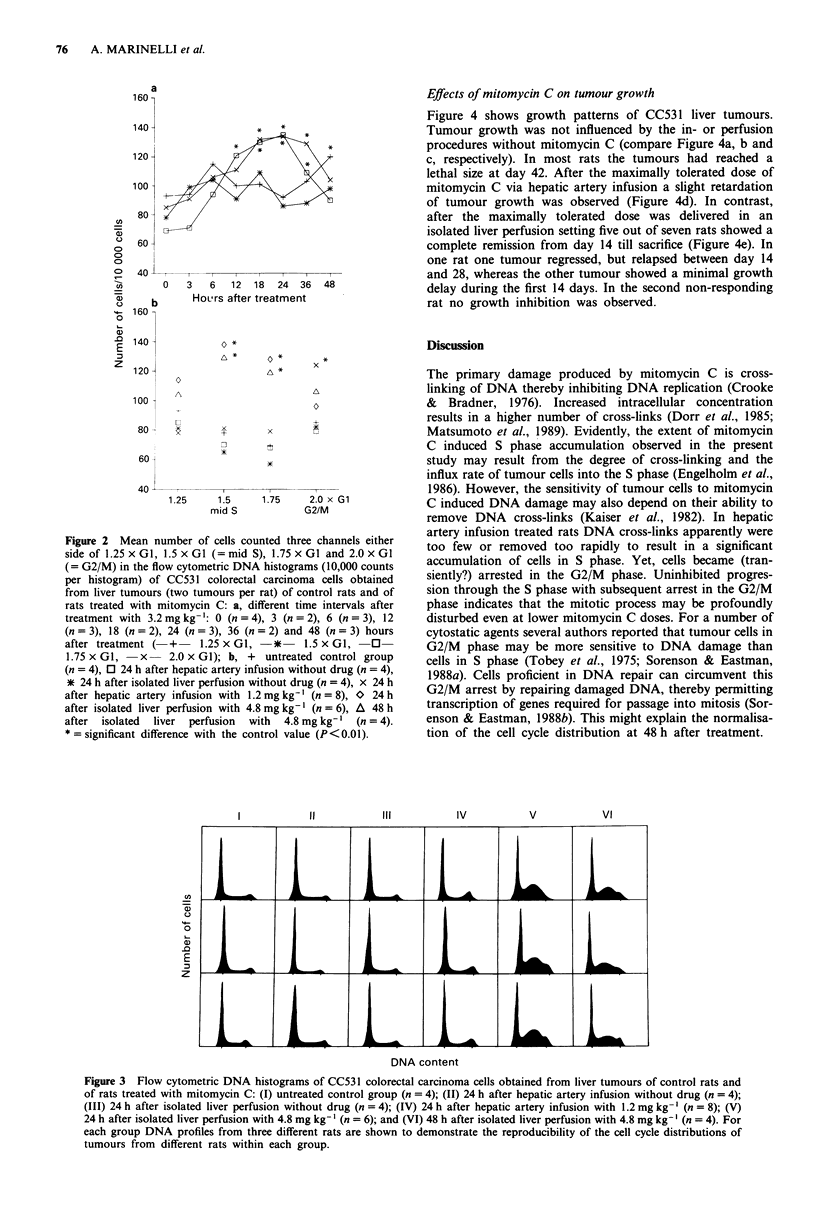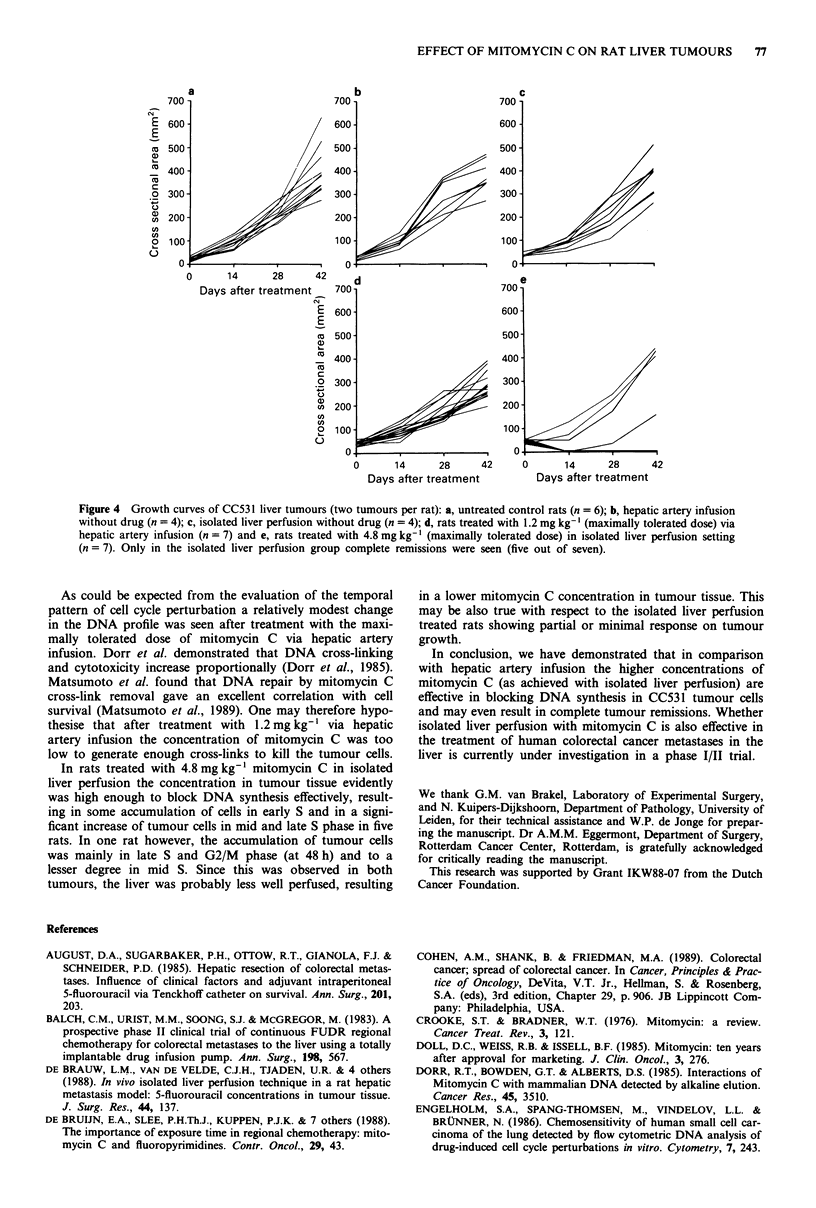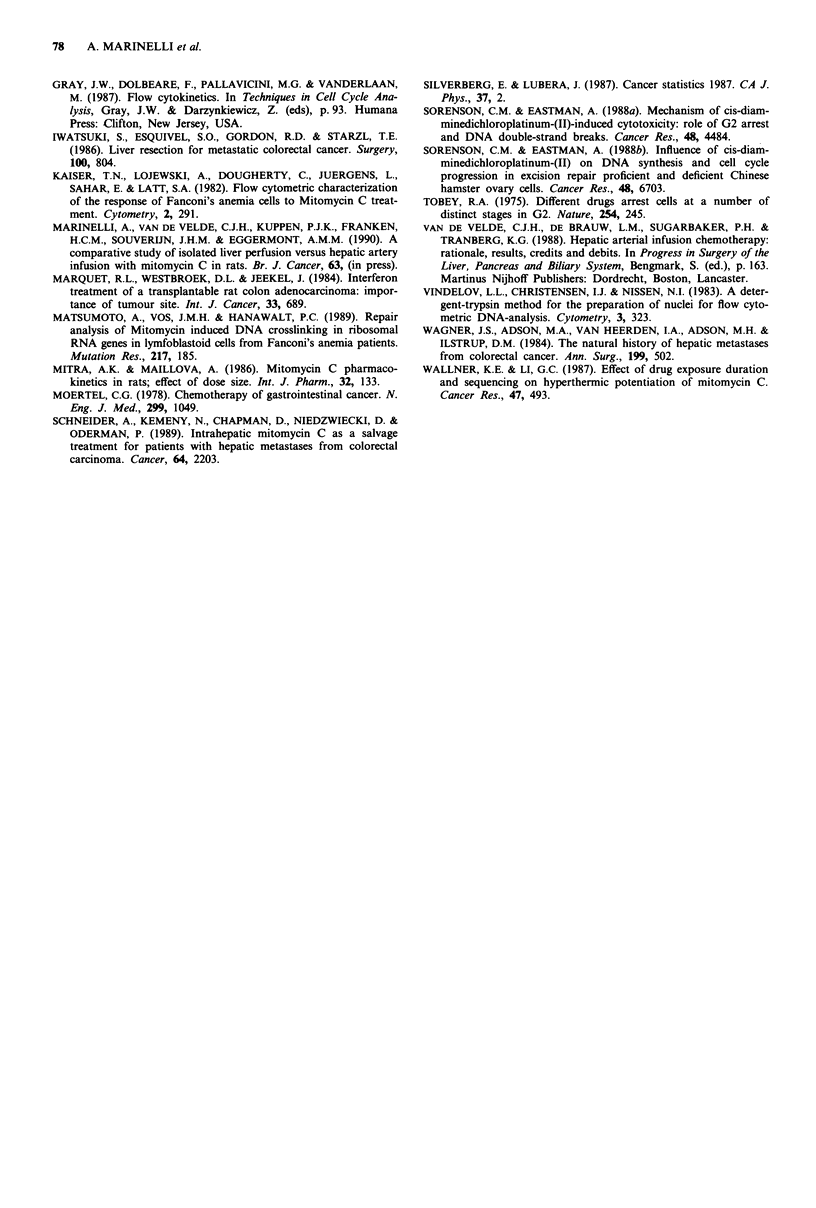# Effectiveness of isolated liver perfusion with mitomycin C in the treatment of liver tumours of rat colorectal cancer.

**DOI:** 10.1038/bjc.1991.242

**Published:** 1991-07

**Authors:** A. Marinelli, F. R. Dijkstra, J. H. van Dierendonck, P. J. Kuppen, C. J. Cornelisse, C. J. van de Velde

**Affiliations:** Department of Surgery, University Hospital, Leiden, The Netherlands.

## Abstract

Dose limiting systemic toxicity prevents sufficient exploitation of the steep dose response relationship of most anticancer agents. In our rat liver tumour model (the CC531 colorectal carcinoma), isolated liver perfusion allows administration of higher doses of mitomycin C than hepatic artery infusion, while systemic toxicity remains minimal. To determine the temporal pattern of mitomycin C induced cytokinetic changes, we analysed flow cytometric DNA histograms of CC531 liver tumours from rats treated with high dose mitomycin C (3.2 mg kg-1) via hepatic artery infusion and sacrificed at different time intervals after treatment. Between 12 and 36 h after treatment, the fraction of cells in late S and G2/M phase had markedly increased. The effects of administration of the respective maximally tolerated doses of mitomycin C in isolated liver perfusion and via hepatic artery infusion on progression of tumour cells through the cell cycle and on gross tumour growth were compared. Isolated liver perfusion with mitomycin C resulted in a significant increase in the proportion of cells in mid and late S, and in some accumulation of cells in early S and G2/M phase at 24 and 48 h after treatment. In contrast, after hepatic artery infusion a significant increase of the fraction of cells in G2/M phase was observed at 24 h after treatment. Monitoring tumour growth after isolated liver perfusion five out of seven rats showed a complete tumour remission, while after hepatic artery infusion only a minimal growth delay was detected. This study demonstrates that isolated liver perfusion in the rat CC531 liver tumour model allows the administration of a well-tolerated dose of mitomycin C being high enough to induce a marked DNA synthesis inhibition and even complete tumour remission.


 © Macmillan Press Ltd., 1991

### Effectiveness of isolated liver perfusion with mitomycin C in the treatment of liver tumours of rat colorectal cancer

A. Marinelli[1], F.R. Dijkstra[1], J.H. van Dierendonck[1], P.J.K. Kuppen[2], C.J. Cornelisse[2] & C.J.H. van de Velde[1,2]

*Departments of [1]Surgery and [2]Pathology, University Hospital, PO Box 9600, 2300 RC Leiden, The Netherlands.*

**Summary** Dose limiting systemic toxicity prevents sufficient exploitation of the steep dose response relationship of most anticancer agents. In our rat liver tumour model (the CC531 colorectal carcinoma), isolated liver perfusion allows administration of higher doses of mitomycin C than hepatic artery infusion, while systemic toxicity remains minimal. To determine the temporal pattern of mitomycin C induced cytokinetic changes, we analysed flow cytometric DNA histograms of CC531 liver tumours from rats treated with high dose mitomycin C (3.2 mg $kg^{-1}$) via hepatic artery infusion and sacrificed at different time intervals after treatment. Between 12 and 36 h after treatment, the fraction of cells in late S and G2/M phase had markedly increased. The effects of administration of the respective maximally tolerated doses of mitomycin C in isolated liver perfusion and via hepatic artery infusion on progression of tumour cells through the cell cycle and on gross tumour growth were compared. Isolated liver perfusion with mitomycin C resulted in a significant increase in the proportion of cells in mid and late S, and in some accumulation of cells in early S and G2/M phase at 24 and 48 h after treatment. In contrast, after hepatic artery infusion a significant increase of the fraction of cells in G2/M phase was observed at 24 h after treatment. Monitoring tumour growth after isolated liver perfusion five out of seven rats showed a complete tumour remission, while after hepatic artery infusion only a minimal growth delay was detected. This study demonstrates that isolated liver perfusion in the rat CC531 liver tumour model allows the administration of a well-tolerated dose of mitomycin C being high enough to induce a marked DNA synthesis inhibition and even complete tumour remission.

Colorectal cancer is one of the most common malignant tumours (Silverberg & Lubera, 1987). The liver is the major site of metastatic spread and in 40% of the patients also the sole site of initial recurrence (Cohen *et al.*, 1989, pp. 906). If the liver metastases are resectable, resection results in a 5 year survival rate of about 35% (August *et al.*, 1985; Iwatsuki *et al.*, 1986). If not resectable the 5 year survival rate with untreated hepatic metastases is less than 5% (Wagner *et al.*, 1984). These patients are eligible for regional therapies. The goal of regional cancer chemotherapy is to obtain high local concentrations of antitumour agents, while maintaining relatively low drug concentrations in the systemic circulation; inasmuch as dose-response curves of most cytostatic agents are steep, exposure of tumour cells to higher concentrations may have a significant impact on the effectiveness of treatment.

Hepatic artery infusion with fluoropyrimidines, currently the most effective drugs in colorectal cancer treatment (Moertel, 1978) met with some success (Balch *et al.*, 1983) albeit with considerable dose limiting morbidity (van de Velde *et al.*, 1988, pp 163). In order to test the applicability of isolated liver perfusion for dose escalation of a variety of drugs, a perfusion technique was developed (de Brauw *et al.*, 1988) in the rat. In this study, we evaluated the effect of mitomycin C, which has a steep dose-response relationship (Mitra & Maillova, 1986; Wallner & Li, 1987; de Bruijn *et al.*, 1988) and has been reported to be a promising drug for colorectal cancer (Doll *et al.*, 1985; Schneider *et al.*, 1989). In a previous study (Marinelli *et al.*, 1990), it was determined that in isolated liver perfusion a four times higher dose of mitomycin C could be administered than via hepatic artery infusion, resulting in a five times higher concentration in tumour tissue. In the present study, we investigated whether this higher tissue concentration is also more effective in the actual elimination of tumour cells.

Correspondence: C.J.H. van de Velde.
Received 10 December 1990; and in revised form 25 February 1991.

Since changes in cell cycle progression are a sensitive indicator for the intracellular effects of cytostatic drugs (Gray *et al.*, 1987, pp. 93), we evaluated changes in DNA distributions of mitomycin C treated tumours by flow cytometry. Effects on tumour growth were studied by sequential caliper measurements.

### Materials and methods

### *Tumour model*

The WAG rat tumour cell line CC531 is a dimethyl-hydrazine induced carcinoma of the colon and is weakly immunogenic (Marquet *et al.*, 1984). Cells are maintained in culture in RPMI 1640 (Dutch modification; GIBCO Europe B.V., Breda, The Netherlands), supplemented with 10% foetal calf serum (GIBCO Limited, Paisley, Scotland), 2 mM L-glutamine, 50 $\mu g\ ml^{-1}$ streptomycin and 50 IU $ml^{-1}$ penicillin. Male WAG/Ola rats weighing 250-300 g were inoculated with cells from cultures between passages 105-115. For *in vivo* inoculation, exponentially growing cells were collected by trypsinisation. The rats underwent laparotomy and $5.10^5$ cells in 0.05 ml Hanks Balanced Salt Solution (University Hospital, Leiden, The Netherlands) were subcapsularly injected in the right and left main lobe of the liver. These rats had tumours at both sites of inoculation without extrahepatic tumour growth. The mean cross sectional area of these tumours ($\pi \times 0.25 \times$ largest diameter $\times$ perpendicular diameter) on day 10 was $37 \pm 13\ mm^2$.

### *Surgical procedures*

Isolated liver perfusion and hepatic artery infusion are described elsewhere (Marinelli *et al.*, 1990). Briefly: in the *isolated liver perfusion* two inflow limbs of the isolated circuit were established by cannulation of the pyloric branch of the portal vein and the gastroduodenal branch of the common hepatic artery. The outflow limb was a cannula inserted in the caval vein via a venotomy just above the right renal vein. Isolation of the liver was achieved by clamping the caval vein, just beneath the diaphragm and just above the right renal vein, the aorta above the coeliac axis and the common hepatic artery plus portal vein. The flow into the portal vein was 20 ml $min^{-1}$ and 4.5 ml $min^{-1}$ into the hepatic artery. The drug was injected as a bolus in the isolated circuit. Recirculation of the perfusate in the *in vivo* perfusion was 25 min. At the end of the ILP procedure a washout was performed with 8 ml saline of 37°C, which was perfused through the liver using the pyloric vein cannula only. Total operation time was 2.0–2.5 h. *Hepatic artery infusion* was performed via the cannulated gastroduodenal branch of the common hepatic artery with its tip in the hepatic artery. During the bolus infusion the common hepatic artery was clamped to prevent retrograde flow into the coeliac axis and aorta. Total operation time was 30–40 min.

*Flow cytometry*

To determine the temporal patterns of mitomycin C induced changes in DNA distributions of hepatic CC531 tumours shortly after the onset of treatment, a series of 21 tumour bearing rats was treated with high dose of mitomycin C (Kyowa Hakko Kogyo Co. Ltd, Tokyo, Japan) via hepatic artery infusion. At respectively 3, 6, 12, 18, 24, 36 and 48 h rats were sacrificed and tumour samples were analysed by flow cytometry.

Dose response patterns were studied by comparing the effect of the respective maximally tolerated dose of mitomycin C as delivered by hepatic artery infusion and isolated liver perfusion. In this study, 26 rats with two tumours each were randomly assigned to five groups that were sacrificed 24 h after treatment: (1) untreated control ($n$ = 4); (2) HAI without drug ($n$ = 4); (3) ILP without drug ($n$ = 4); (4) 1.2 mg $kg^{-1}$ MMC by HAI ($n$ = 8) and; (5) 4.8 mg $kg^{-1}$ by ILP ($n$ = 6). Additionally, rats were sacrificed 48 h after ILP with 4.8 mg $kg^{-1}$ MMC ($n$ = 4).

Immediately after sacrifice, both tumours were excised, finely minced, diluted in citrate buffer (0.04 M, pH 7.6) with 5% dimethylsulfoxide (Vindelov *et al.*, 1983), and frozen at −70°C. Prior to analysis the samples were thawed and centrifugated at 2,000 r.p.m. for 15 min. Suspensions of single nuclei were prepared according to the detergent-trypsin procedure described by Vindelov *et al.* (1983), and stained with propidium iodide (Sigma, St Louis, MO).

Samples were analysed on a FACScan flow cytometer (Becton & Dickinson, Mountain View, CA). Propidium iodide (PI) fluorescence was excited at 488 nm and measured at 585 nm. Cell cycle distributions were calculated with aid of the CellFIT Software using the Sum of Broadened Rectangles (SOBR) model. However, this model as well as the R-fit and S-fit model gave erratic results for the highly distorted DNA distributions in part of the experiments. For this reason, histograms are presented without calculations of the percentages of cells in the different cell cycle compartments. However, cells were counted three channels either side of 1.25 × G1, 1.5 × G1 (= mid S), 1.75 × G1 and 2.0 × G1 (= G2/M) to achieve a more objective evidence on accumulation of cells in S and G2/M phase.

*Tumour growth*

To study the effects of mitomycin C on tumour growth, tumour bearing rats (two tumours each) were randomly assigned to: (1) untreated control ($n$ = 6); (2) hepatic artery infusion without drug ($n$ = 4); (3) isolated liver perfusion without drug ($n$ = 4); (4) hepatic artery infusion with 1.2 mg $kg^{-1}$ ($n$ = 7), and (5) isolated liver perfusion with 4.8 mg $kg^{-1}$ ($n$ = 7). On days 0 (day of treatment), 14, 28 and 42 rats were weighed and in order to measure liver tumours laparotomy was performed. Cross sectional tumour areas were estimated by caliper measurements and calculated as: $\pi \times 0.25 \times$ length $\times$ width.

*Statistical evaluation*

Statistical significance was determined by one-way analysis of variance using the SPSS package. To compare each treatment group with the control group the option contrast was used with separate variance estimates. A $P$ value $<0.01$ was selected to denote statistical significance between groups.

### Results

*Effects of mitomycin C on cell cycle distribution*

Flow cytometric DNA histograms of CC531 liver tumours at different time intervals after 3.2 mg $kg^{-1}$ mitomycin C (as delivered by hepatic artery infusion) revealed a significant increase of the proportion of cells in the late S and G2/M phase between 12 and 36 h after treatment (Figures 1 and 2a). At 48 h, the histograms closely resembled those of the control group (baseline values: G0/G1: 64 ± 3%, S: 30 ± 4% and G2/M: 6 ± 2%). Therefore, 24 h was chosen as time interval for the evaluation of dose response relationships.

Histograms obtained with tumour cells collected after hepatic artery infusion or isolated liver perfusion without drug (Figures 2b, 3 II and III, respectively) were identical to those obtained from tumours of untreated control rats (Figures 2b and 3 I). After treatment with 1.2 mg $kg^{-1}$ via hepatic artery infusion, the proportion of cells in G2/M phase was significantly increased (Figures 2b and 3 IV). However, in late S phase no accumulation of cells was seen after treatment with 1.2 mg $kg^{-1}$, while late S was significantly increased after treatment with 3.2 mg $kg^{-1}$ (Figures 1, 2a and b). Treatment with 4.8 mg $kg^{-1}$ mitomycin C in isolated liver perfusion resulted in a significant accumulation of tumour cells in mid and late S phase and also in some accumulation in early S and in G2/M phase (Figures 2b and 3 V). Forty-eight hours after treatment mid and late S phase were still significantly increased and there was still an accumulation of cells in early S and in G2/M phase (Figures 2b and 3 VI).

**Figure 1** Flow cytometric DNA histograms of CC531 colorectal carcinoma cells treated with 3.2 mg $kg^{-1}$ via hepatic artery infusion and obtained from liver tumours at different time intervals after treatment: 0, 3, 6, 12, 18, 24, 36 and 48 h after treatment. For each time interval, histograms of tumours from two different rats are shown.

**Figure 2** Mean number of cells counted three channels either side of 1.25 × G1, 1.5 × G1 (= mid S), 1.75 × G1 and 2.0 × G1 (= G2/M) in the flow cytometric DNA histograms (10,000 counts per histogram) of CC531 colorectal carcinoma cells obtained from liver tumours (two tumours per rat) of control rats and of rats treated with mitomycin C: **a**, different time intervals after treatment with 3.2 mg kg$^{-1}$: 0 ($n = 4$), 3 ($n = 2$), 6 ($n = 3$), 12 ($n = 3$), 18 ($n = 2$), 24 ($n = 3$), 36 ($n = 2$) and 48 ($n = 3$) hours after treatment (—+— 1.25 × G1, —✱— 1.5 × G1, —□— 1.75 × G1, —×— 2.0 × G1); **b**, + untreated control group ($n = 4$), □ 24 h after hepatic artery infusion without drug ($n = 4$), ✱ 24 h after isolated liver perfusion without drug ($n = 4$), × 24 h after hepatic artery infusion with 1.2 mg kg$^{-1}$ ($n = 8$), ◇ 24 h after isolated liver perfusion with 4.8 mg kg$^{-1}$ ($n = 6$), △ 48 h after isolated liver perfusion with 4.8 mg kg$^{-1}$ ($n = 4$). * = significant difference with the control value ($P < 0.01$).

*Effects of mitomycin C on tumour growth*

Figure 4 shows growth patterns of CC531 liver tumours. Tumour growth was not influenced by the in- or perfusion procedures without mitomycin C (compare Figure 4a, b and c, respectively). In most rats the tumours had reached a lethal size at day 42. After the maximally tolerated dose of mitomycin C via hepatic artery infusion a slight retardation of tumour growth was observed (Figure 4d). In contrast, after the maximally tolerated dose was delivered in an isolated liver perfusion setting five out of seven rats showed a complete remission from day 14 till sacrifice (Figure 4e). In one rat one tumour regressed, but relapsed between day 14 and 28, whereas the other tumour showed a minimal growth delay during the first 14 days. In the second non-responding rat no growth inhibition was observed.

### Discussion

The primary damage produced by mitomycin C is cross-linking of DNA thereby inhibiting DNA replication (Crooke & Bradner, 1976). Increased intracellular concentration results in a higher number of cross-links (Dorr et al., 1985; Matsumoto et al., 1989). Evidently, the extent of mitomycin C induced S phase accumulation observed in the present study may result from the degree of cross-linking and the influx rate of tumour cells into the S phase (Engelholm et al., 1986). However, the sensitivity of tumour cells to mitomycin C induced DNA damage may also depend on their ability to remove DNA cross-links (Kaiser et al., 1982). In hepatic artery infusion treated rats DNA cross-links apparently were too few or removed too rapidly to result in a significant accumulation of cells in S phase. Yet, cells became (transiently?) arrested in the G2/M phase. Uninhibited progression through the S phase with subsequent arrest in the G2/M phase indicates that the mitotic process may be profoundly disturbed even at lower mitomycin C doses. For a number of cytostatic agents several authors reported that tumour cells in G2/M phase may be more sensitive to DNA damage than cells in S phase (Tobey et al., 1975; Sorenson & Eastman, 1988*a*). Cells proficient in DNA repair can circumvent this G2/M arrest by repairing damaged DNA, thereby permitting transcription of genes required for passage into mitosis (Sorenson & Eastman, 1988*b*). This might explain the normalisation of the cell cycle distribution at 48 h after treatment.

**Figure 3** Flow cytometric DNA histograms of CC531 colorectal carcinoma cells obtained from liver tumours of control rats and of rats treated with mitomycin C: (I) untreated control group ($n = 4$); (II) 24 h after hepatic artery infusion without drug ($n = 4$); (III) 24 h after isolated liver perfusion without drug ($n = 4$); (IV) 24 h after hepatic artery infusion with 1.2 mg kg$^{-1}$ ($n = 8$); (V) 24 h after isolated liver perfusion with 4.8 mg kg$^{-1}$ ($n = 6$); and (VI) 48 h after isolated liver perfusion with 4.8 mg kg$^{-1}$ ($n = 4$). For each group DNA profiles from three different rats are shown to demonstrate the reproducibility of the cell cycle distributions of tumours from different rats within each group.

**Figure 4** Growth curves of CC531 liver tumours (two tumours per rat): **a**, untreated control rats ($n = 6$); **b**, hepatic artery infusion without drug ($n = 4$); **c**, isolated liver perfusion without drug ($n = 4$); **d**, rats treated with 1.2 mg kg$^{-1}$ (maximally tolerated dose) via hepatic artery infusion ($n = 7$) and **e**, rats treated with 4.8 mg kg$^{-1}$ (maximally tolerated dose) in isolated liver perfusion setting ($n = 7$). Only in the isolated liver perfusion group complete remissions were seen (five out of seven).

As could be expected from the evaluation of the temporal pattern of cell cycle perturbation a relatively modest change in the DNA profile was seen after treatment with the maximally tolerated dose of mitomycin C via hepatic artery infusion. Dorr *et al.* demonstrated that DNA cross-linking and cytotoxicity increase proportionally (Dorr *et al.*, 1985). Matsumoto *et al.* found that DNA repair by mitomycin C cross-link removal gave an excellent correlation with cell survival (Matsumoto *et al.*, 1989). One may therefore hypothesise that after treatment with 1.2 mg kg$^{-1}$ via hepatic artery infusion the concentration of mitomycin C was too low to generate enough cross-links to kill the tumour cells.

In rats treated with 4.8 mg kg$^{-1}$ mitomycin C in isolated liver perfusion the concentration in tumour tissue evidently was high enough to block DNA synthesis effectively, resulting in some accumulation of cells in early S and in a significant increase of tumour cells in mid and late S phase in five rats. In one rat however, the accumulation of tumour cells was mainly in late S and G2/M phase (at 48 h) and to a lesser degree in mid S. Since this was observed in both tumours, the liver was probably less well perfused, resulting in a lower mitomycin C concentration in tumour tissue. This may be also true with respect to the isolated liver perfusion treated rats showing partial or minimal response on tumour growth.

In conclusion, we have demonstrated that in comparison with hepatic artery infusion the higher concentrations of mitomycin C (as achieved with isolated liver perfusion) are effective in blocking DNA synthesis in CC531 tumour cells and may even result in complete tumour remissions. Whether isolated liver perfusion with mitomycin C is also effective in the treatment of human colorectal cancer metastases in the liver is currently under investigation in a phase I/II trial.

We thank G.M. van Brakel, Laboratory of Experimental Surgery, and N. Kuipers-Dijkshoorn, Department of Pathology, University of Leiden, for their technical assistance and W.P. de Jonge for preparing the manuscript. Dr A.M.M. Eggermont, Department of Surgery, Rotterdam Cancer Center, Rotterdam, is gratefully acknowledged for critically reading the manuscript.

This research was supported by Grant IKW88-07 from the Dutch Cancer Foundation.

### References

AUGUST, D.A., SUGARBAKER, P.H., OTTOW, R.T., GIANOLA, F.J. & SCHNEIDER, P.D. (1985). Hepatic resection of colorectal metastases. Influence of clinical factors and adjuvant intraperitoneal 5-fluorouracil via Tenckhoff catheter on survival. *Ann. Surg.*, **201**, 203.

BALCH, C.M., URIST, M.M., SOONG, S.J. & MCGREGOR, M. (1983). A prospective phase II clinical trial of continuous FUDR regional chemotherapy for colorectal metastases to the liver using a totally implantable drug infusion pump. *Ann. Surg.*, **198**, 567.

DE BRAUW, L.M., VAN DE VELDE, C.J.H., TJADEN, U.R. & 4 others (1988). *In vivo* isolated liver perfusion technique in a rat hepatic metastasis model: 5-fluorouracil concentrations in tumour tissue. *J. Surg. Res.*, **44**, 137.

DE BRUIJN, E.A., SLEE, P.H.Th.J., KUPPEN, P.J.K. & 7 others (1988). The importance of exposure time in regional chemotherapy: mitomycin C and fluoropyrimidines. *Contr. Oncol.*, **29**, 43.

COHEN, A.M., SHANK, B. & FRIEDMAN, M.A. (1989). Colorectal cancer; spread of colorectal cancer. In *Cancer, Principles & Practice of Oncology*, DeVita, V.T. Jr., Hellman, S. & Rosenberg, S.A. (eds), 3rd edition, Chapter 29, p. 906. JB Lippincott Company: Philadelphia, USA.

CROOKE, S.T. & BRADNER, W.T. (1976). Mitomycin: a review. *Cancer Treat. Rev.*, **3**, 121.

DOLL, D.C., WEISS, R.B. & ISSELL, B.F. (1985). Mitomycin: ten years after approval for marketing. *J. Clin. Oncol.*, **3**, 276.

DORR, R.T., BOWDEN, G.T. & ALBERTS, D.S. (1985). Interactions of Mitomycin C with mammalian DNA detected by alkaline elution. *Cancer Res.*, **45**, 3510.

ENGELHOLM, S.A., SPANG-THOMSEN, M., VINDELOV, L.L. & BRÜNNER, N. (1986). Chemosensitivity of human small cell carcinoma of the lung detected by flow cytometric DNA analysis of drug-induced cell cycle perturbations *in vitro*. *Cytometry*, **7**, 243.

GRAY, J.W., DOLBEARE, F., PALLAVICINI, M.G. & VANDERLAAN, M. (1987). Flow cytokinetics. In *Techniques in Cell Cycle Analysis*, Gray, J.W. & Darzynkiewicz, Z. (eds), p. 93. Humana Press: Clifton, New Jersey, USA.

IWATSUKI, S., ESQUIVEL, S.O., GORDON, R.D. & STARZL, T.E. (1986). Liver resection for metastatic colorectal cancer. *Surgery*, **100**, 804.

KAISER, T.N., LOJEWSKI, A., DOUGHERTY, C., JUERGENS, L., SAHAR, E. & LATT, S.A. (1982). Flow cytometric characterization of the response of Fanconi's anemia cells to Mitomycin C treatment. *Cytometry*, **2**, 291.

MARINELLI, A., VAN DE VELDE, C.J.H., KUPPEN, P.J.K., FRANKEN, H.C.M., SOUVERIJN, J.H.M. & EGGERMONT, A.M.M. (1990). A comparative study of isolated liver perfusion versus hepatic artery infusion with mitomycin C in rats. *Br. J. Cancer*, **63**, (in press).

MARQUET, R.L., WESTBROEK, D.L. & JEEKEL, J. (1984). Interferon treatment of a transplantable rat colon adenocarcinoma: importance of tumour site. *Int. J. Cancer*, **33**, 689.

MATSUMOTO, A., VOS, J.M.H. & HANAWALT, P.C. (1989). Repair analysis of Mitomycin induced DNA crosslinking in ribosomal RNA genes in lymfoblastoid cells from Fanconi's anemia patients. *Mutation Res.*, **217**, 185.

MITRA, A.K. & MAILLOVA, A. (1986). Mitomycin C pharmacokinetics in rats; effect of dose size. *Int. J. Pharm.*, **32**, 133.

MOERTEL, C.G. (1978). Chemotherapy of gastrointestinal cancer. *N. Eng. J. Med.*, **299**, 1049.

SCHNEIDER, A., KEMENY, N., CHAPMAN, D., NIEDZWIECKI, D. & ODERMAN, P. (1989). Intrahepatic mitomycin C as a salvage treatment for patients with hepatic metastases from colorectal carcinoma. *Cancer*, **64**, 2203.

SILVERBERG, E. & LUBERA, J. (1987). Cancer statistics 1987. *CA J. Phys.*, **37**, 2.

SORENSON, C.M. & EASTMAN, A. (1988*a*). Mechanism of cis-diamminedichloroplatinum-(II)-induced cytotoxicity: role of G2 arrest and DNA double-strand breaks. *Cancer Res.*, **48**, 4484.

SORENSON, C.M. & EASTMAN, A. (1988*b*). Influence of cis-diamminedichloroplatinum-(II) on DNA synthesis and cell cycle progression in excision repair proficient and deficient Chinese hamster ovary cells. *Cancer Res.*, **48**, 6703.

TOBEY, R.A. (1975). Different drugs arrest cells at a number of distinct stages in G2. *Nature*, **254**, 245.

VAN DE VELDE, C.J.H., DE BRAUW, L.M., SUGARBAKER, P.H. & TRANBERG, K.G. (1988). Hepatic arterial infusion chemotherapy: rationale, results, credits and debits. In *Progress in Surgery of the Liver, Pancreas and Biliary System*, Bengmark, S. (ed.), p. 163. Martinus Nijhoff Publishers: Dordrecht, Boston, Lancaster.

VINDELOV, L.L., CHRISTENSEN, I.J. & NISSEN, N.I. (1983). A detergent-trypsin method for the preparation of nuclei for flow cytometric DNA-analysis. *Cytometry*, **3**, 323.

WAGNER, J.S., ADSON, M.A., VAN HEERDEN, I.A., ADSON, M.H. & ILSTRUP, D.M. (1984). The natural history of hepatic metastases from colorectal cancer. *Ann. Surg.*, **199**, 502.

WALLNER, K.E. & LI, G.C. (1987). Effect of drug exposure duration and sequencing on hyperthermic potentiation of mitomycin C. *Cancer Res.*, **47**, 493.